# A qualitative exploration of obesity bias and stigma in Irish healthcare; the patients’ voice

**DOI:** 10.1371/journal.pone.0260075

**Published:** 2021-11-29

**Authors:** Grainne O’Donoghue, Caitriona Cunningham, Melvina King, Chantel O’Keefe, Andrew Rofaeil, Sinead McMahon

**Affiliations:** School of Public Health, Physiotherapy and Sports Science, University College Dublin, Dublin, Ireland; Mayo Clinic Rochester: Mayo Clinic Minnesota, UNITED STATES

## Abstract

**Background:**

Current data indicates 70% of adults with obesity report experiencing bias and stigmatisation when engaging with healthcare. Most studies to date, have focused on weight bias from a healthcare professional’s perspective. Few have explored weight bias from the perspective of the individual living with obesity and no study has conducted this research in the Irish context.

**Aims:**

This study explored, the lived-in experience of individuals afflicted with obesity, when interacting with the Irish healthcare system. It examined whether participants encountered weight bias and stigma, if so, how it may have impacted them and gathered their suggestions on how it could be best addressed.

**Methods:**

Employing a phenomenological approach, purposive sampling and semi-structured interviews were conducted with 15 individuals living with class II (BMI 35.0–39.9) or III obesity (BMI *≥*40kg/m^2^) who reported regular and consistent engagement with the Irish healthcare system. Predominant emergent themes were categorised using the interview domains; (1) experiences of obesity bias and stigma, (2) impact of this bias and stigma and (3) suggested avenues to reduce bias and stigma.

**Findings:**

Participants reported experiencing high levels of weight bias and stigmatisation. Relating to experiences, three themes were identified; interpersonal communication, focus of care and physical environment. In terms of its impact, there were two emergent themes; negativity towards future healthcare and escalation of unhealthy behaviours. Suggested avenues to eliminate bias and stigma included the introduction of a timely and clear clinical pathway for obesity management and a focus on HCPs education in relation to obesity causes and complexity.

**Conclusions:**

Outside of specialist obesity tertiary care, weight bias and stigmatisation is commonly reported in the Irish healthcare system. It is a significant issue for those living with obesity, detrimental to their physiological and psychological health. A concerted effort by HCPs across clinical, research and educational levels is required to alleviate its harmful effects.

## Introduction

Obesity is considered to be one of the most critical problems in our society, so much so that it is now recognised as a chronic disease as well as being referred to as a public health epidemic [1]. In recent decades, global prevalence of individuals who meet the criteria of being classified as obese has increased at an astonishing rate. Along with the rest of the world, Ireland has experienced an increase in obesity, and is predicted to become the most obese country in Europe by 2025 [2]. According to the annual Irish Department of Health survey, last conducted in 2019, over 20% of the Irish population are classified as obese; with an expectation that this figure will rise to over 40% by 2030 [3].

As the obesity epidemic increases, so too, does weight bias and stigma. Weight bias is defined as negative attitudes towards, and beliefs about others because of their weight [4] and can lead to obesity stigma, which is the social sign or label affixed to an individual who is the victim of prejudice [5]. Unfortunately, health care professionals (HCPs) are not immune to this bias; even those specialising in the field of obesity have been shown to both endorse and display weight bias at an alarming frequency [6]. Current data indicates up to 70% of adults with obesity report experiencing stigmatisation in the healthcare setting [7]. Existing literature reports that physicians and other healthcare professionals perceive people with obesity as undisciplined, lazy, weak-willed and unlikely to comply with treatment or make lifestyle changes [8–10]. These assumptions have been shown to result in multiple negative encounters for patients living with obesity, at the hands of their healthcare provider [11]. This is problematic on many levels, not least, because it has been shown to result in people with obesity not seeking healthcare, having reduced confidence in HCPs and poor treatment outcomes [12–14].

Despite increasing attention to this issue in the scientific and clinical community, very few studies have explored weight bias from the perspective of individuals themselves, especially in healthcare environments. Those that have, have examined the patients’ overall experience, without specifically exploring weight bias and stigma [15] or have limited the research to a specific healthcare setting, such as primary care [16, 17] or maternity services [18, 19]. To the best of our knowledge, no studies, to date, have investigated the patients’ experiences of weight bias and stigma across primary, secondary and tertiary healthcare, nor have examined how this bias and stigma has impacted patients’ health and wellbeing.

### Study aims

Therefore the overall aim of this study was to explore, using a qualitative approach, the lived-in experience of patients living with obesity. The central research question is ‘what is the Irish healthcare experience like for people living with obesity?’ The associated sub-questions are (a) how do people living with obesity experience this weight bias and stigma?, (b) in what way does it impact their health and wellbeing and (c) can they identify avenues for change and development to address this bias, with the ultimate goal of improving their healthcare experiences [20].

## Methods

The Standards for Reporting Qualitative Research (SRQR) 21 items checklist was used to ensure transparency in this research from initial design through to manuscript completion [21] (S1 Table).

### Study design

To construct a rich understanding of the lived healthcare experiences of obesity bias and stigma among patients, we used a phenomenological approach. This is an approach to qualitative research with an idiographic focus, which means that it aims to offer insights into how a person, in a given context, makes sense of a given phenomenon. Usually, these phenomena relate to experiences of some personal significance [22–24]. We used semi-structured interviews to gather participants’ personal experiences of weight bias and stigma in the Irish healthcare setting. This methodology has been reported to be wholly appropriate for phenomenology as it provides the basis of getting respondents to describe experience, helps manage the process of questioning and affords clarity [25]. Questions were generally broad and open ended so that participants had sufficient opportunity to express their view point extensively and were asked in the vocabulary of the individual being interviewed. A detailed interview guide is provided in S1 File

### Research team

Experienced qualitative researchers SMcM (PhD) and GO’D (PhD), academic staff members in the UCD School of Public Health, Physiotherapy and Sports Science conducted the interviews and oversaw the analysis. Both SMcM and GO’D are healthcare professionals and have conducted several previous studies utilising phenomenology and qualitative methods (focus groups, semi-structured interviews). Another member of the academic staff (CC) and three MSc health professional students (MK, CO’K and AR) transcribed the interview data and assisted in the analyses.

### Participants and recruitment

A purposive sampling strategy was employed as it has been identified as the most important kind of non-probability sampling, when seeking participants who have had experiences relating to the phenomenon to be researched [21]. An a priori sample size of 12 to 15 was selected based on recommendations for qualitative studies [26] and the fact that data saturation is not normally an aim in phenomenological analysis, owing to the concern to obtain ‘full and rich personal accounts’, which highlights the particular analytical focus within individual accounts in this approach [27].

Based on the World Health Organization body mass index (BMI) classification, people living with class II (BMI 35.0–39.9kg/m^2^; moderate health risk) or class III (BMI *≥*40kg/m^2^; high health risk) obesity [28] having had extensive experience (>10 years of regular contact (>3 episodes per year) of primary, secondary and tertiary healthcare in the Republic of Ireland were invited to participate. Primary care was defined as general medical practice, secondary care as hospital care (e.g., in-patient, or out-patients orthopedics) and tertiary care as specialist weight management/obesity care, specifically, the National Weight Management Service.

Initial contact with potential participants was made via the Patient Representative of the Association for the Study of Obesity (ASOI) in Ireland, in October 2019. The ASOI patient representative distributed the study flyer at group meetings and posted it on related social media platforms. If interested in participating, potential participants were requested to contact the researchers (GO’D, SMcM) directly via email or telephone. They were then provided with a verbal study overview and given access to the study information and consent documentation online or via hardcopy. Written informed consent was obtained and due to the sensitive nature of the topic, it was reiterated to participants prior to interview, that all data would be de-identified, amalgamated and reported anonymously to ensure privacy and confidentiality.

Within two weeks of starting recruitment, a total of nineteen potential participants were screened. Four were excluded (two had not attended for specialist weight management/obesity care and two had less than 10 years’ experience of the Irish healthcare system). The remaining fifteen took part in the study, which ran over a six-week period (Nov-Dec 2019).

Of the 15 participants, 13 were women and two men (mean age: 46 years ±10). Self-reported height and weight was used to calculate body mass index (BMI) with four participants categorised as living with class II obesity and 11 with class III (mean BMI: 42.1 ±10.8). More than half of those that participated had undergone bariatric surgery (n = 8) and all 15 had had extensive interaction (>10 years of regular contact with a minimum of 5 contacts per year) between primary, secondary and tertiary healthcare services in the Republic of Ireland.

### Interviews and setting

Fifteen individual semi-structured interviews were conducted by SMcM. All interviews took place privately via telephone from a university office. Each interview lasted approximately one-hour and the interviewers were guided by a number of open-ended questions. A semi-structured interview guide was developed (S1 File), piloted on two volunteers and revised accordingly. Interview topics were broadly focused on (1) participants’ experiences of obesity bias and stigma in healthcare, (2) how these experiences have impacted them and (3) their views on how to address the issue of weight bias and stigma in healthcare. Additional probing or follow-up questions were asked in order to clarify participants’ responses.

### Ethical approval

The study was performed in line with the Helsinki declaration and approved by Human Research Ethics Committee in University College Dublin (LS-19-70-ODonoghue).

### Data analysis

Interviews were transcribed verbatim and entered onto NVivo 12, a qualitative data management software programme. Participants were assigned pseudonyms and all other identifying information such as healthcare practitioners and specific clinic names were removed. The ‘Framework Analysis’ (FA) method was used to analyse the data [29]. This form of analysis is not aligned with a particular epistemological, philosophical, or theoretical approach. Rather it is a flexible tool that can be adapted for use with many qualitative approaches that aim to generate themes. It is appropriate for thematic analysis of textual data, particularly interview transcripts, where it is important to be able to compare and contrast data by themes across many cases, while also situating each perspective in context by retaining the connection to other aspects of each individual’s account. Framework analysis provides five systematic and visible stages (as illustrated in S1 Fig) to the analysis process and allows for a mixed inductive/deductive approach, by including a priori as well as emergent concepts when coding [29]. Cohen’s data statistic was used to explore inter-coder reliability. Both intra-rater (0.9) and inter-rater (0.85) reliability were deemed acceptable [30].

## Results

### Main findings

Predominant emergent themes are presented in relation to the interview domains; (1) experiences of obesity bias and stigma in healthcare, (2) impact of obesity bias and stigma on health and wellbeing and (3) suggested avenue to address this bias and stigma. All results are supported by direct quotations from the interview transcripts. All quotes are referenced with a number from 1 to 15; corresponding to individual participants and the healthcare setting where they originated (PC: primary care, SC: secondary care and TC: tertiary care (weight management)). Table 1 provides an overview of main themes and their sub-themes.

**Table 1:** Table of themes and subthemes.

Themes (Subthemes)	Summary
**1. Experiences of obesity bias and stigma**	
▪ Interpersonal Communication	Verbal and non-verbal communication, including facial micro-expressions of disgust and contempt, accusatory judgemental comments and derogatory language.
▪ Focus of Care	Concerns over medical issues all attributed to obesity and not given due consideration. Presumption among HCPs ^a^ that weight loss would resolve the issue, irrespective of what the symptoms were.
▪ Physical Environment	Experiences with various aspects of clinic or hospital environments, mostly relating to clinic space, furniture, medical equipment, and supplies capacity.
**2. Impact of bias and stigma on health**	
▪ Future Healthcare Interactions	Previous experiences of weight bias in primary and secondary care resulted in missed or cancelled appointments and occasionally, exacerbation of minor ailments to more serious medical issues.
▪ Escalation of Unhealthy Behaviours	Escalation of harmful behaviour related to food, physical inactivity and/or smoking, associated with either personal ambivalence or shame, as a response to being subjected to stigmatisation by a HCP.
**3. Avenues to address bias and stigma**	
▪ Empathy and Equity	Understanding from HCPs as to how people living with obesity feel and using that understanding to guide actions. Be treated with the same level of professionalism or offered the same care as patients without obesity.
▪ Healthcare Professional Education	Formal obesity education at HCPs entry and graduate level, and training for current HCPs, all devised in collaboration with the patient living with obesity.
▪ Obesity Clinical Pathways	Access to a structured and timely clinical pathway with the opportunity to access specialist services, much earlier on the obesity journey.

^a^ Healthcare Professionals.

#### 1. Experiences of obesity bias and stigma in healthcare

Three emergent themes have been identified in relation to participants’ experiences of obesity bias and stigma, the strongest being interpersonal communication, followed by focus of care and the physical environment.

*Interpersonal Communication*. Two sub-themes were identified within this theme: non-verbal and verbal communication. Mostly participants reported negative experiences in relation to communication, particularly in primary care and obstetric clinics. These included facial micro-expressions of disgust and contempt, accusatory judgemental comments and a generalised use of derogatory language that invoked feelings of shame, guilt and embarrassment and for some, serious emotional trauma. Their experience in the tertiary setting (weight management clinic) was almost always positive, with no ambiguous or judgemental non-verbal or verbal communication.

*“No one is completely rude*, *it is much subtler than that*, *it’s the facial expressions when you walk not the room first–no eye contact at all” (s13*, *PC) “I feel my doctor is looking at the computer and making faces*. *I sometimes want to say why are you making that face? Just say what you have to say” (s2*, *PC)*

*“I didn’t realise you were so fat*, *we can’t possibly move someone as heavy as you” (s11*, *SC (obstetrics))*

*“I even had a GP look me up and down and say to me*, *you’d be a very pretty girl if you’d lose some of that weight” (s3*, *PC)*

*“It was a totally different experience the first time I came to ___*, *I was listened too*, *the doctor looked straight at me and I actually felt what I had to say mattered” (s3*, *TC)*

*Focus of Care*. Two sub-themes were identified within the focus of care theme; attributing all health-related problems to weight and refusal of care. In the majority of cases, participants found that their concerns over most medical issue were not addressed seriously and were attributed to their weight and not given due consideration. They were told weight loss would resolve the medical issue, irrespective of what the symptoms were. This was particularly apparent in the primary care setting. Participants felt they were often abruptly dismissed and not referred onwards to specialty services, which in their opinion, were, at times, warranted. In terms of the weight management service, positive comments related to the supportive, unprejudiced consultations from all members of the clinical team and the holistic integrated approach, irrespective of whether the medical issue being discussed was obesity related.

*“Every time I go to the doctor*, *he blames everything on my weight*. *I remember once being told you wouldn’t get such a bad flu if you weren’t so obese” (s2*, *PC)*

*“My fourth pregnancy was twins and I lost one of the babies and the consultant put everything that went wrong down to my weight*, *even losing the baby (s1*, *SC (obstetrics))*.

*“My doctor wouldn’t send me for an x-ray for my knee*. *He told my knee would be fine if I lost 3 stone*. *Twelve weeks later*, *I eventually had an MRI*, *which I had to pay for myself*, *and it showed a cartilage problem and I ended up needed surgery” (s7*, *PC)*.

*Eventually he referred me to orthopaedics and the first thing the orthopaedic surgeon said was the same*, *I needed to lose weight*. *When I did get an x-ray*, *it showed a fracture*. *I’m not completely stupid*, *I know my weight doesn’t help*, *but it wasn’t the reason I broke my leg (s9*, *SC (orthopaedics))*.

*“In the weight management clinic*, *I can ask about any ailment I have*, *and it is not automatically attributed to my weight*. *The team there were the ones that explained to me that I didn’t need to see a surgeon because of my back and suggested I start by seeing the physiotherapist*, *whom they referred me to (s5*, *TC)*.

*Physical Environment*. There were no sub themes identified from this theme. Participants reported inadequacies and negative experiences with various aspects of the clinic or hospital environment, mostly relating to clinic space, furniture, medical equipment and supplies capacity. The described physical environment limitations often resulted in a sense of embarrassment and shame.

*“One time I had to get an MRI scan*. *The machine was very small*. *I didn’t fit in it and the radiographer said you could try another clinic*, *but the really overweight people sometimes have to go to the vet hospital*. *This was about 10 years ago so maybe things have changed but I’m not so sure” (s3*, *SC (radiology))*

*“I was in ___ hospital in a gown that was too small*, *no underwear on*, *feeling like I was on show*, *wondering and hoping that the trolley would hold me” (s1*, *SC (obstetrics))*

*“Your arm is too fat for that blood pressure cuff*. *I’ll have to go and see if I can get the one for bigger arms–and then there is a big commotion (s11*, *PC)*

*“Parking spaces in hospitals are a thing I dread*. *I’ve had a to ask a friend to get into my car to back it out because I can’t get into it because of the limited space if a car parks beside you*. *I have driven around for ever until I can get a corner space so my door can open wide (s9*, *SC)*

#### 2. Impact of bias and stigma on health and wellbeing

Two sub-themes were identified within this theme; feelings towards future healthcare interactions and escalation of unhealthy behaviours associated with personal ambivalence and shame.

*Future Healthcare Interactions*. A number of participants reported a heightened level of sensitivity and negative pre-conceptions in advance of both Primary and Secondary Care appointments. They associated these feelings with their previous experiences of weight bias in these settings. This in turn resulted in them missing /cancelling appointments and on occasion, exacerbation of minor ailments to more serious medical issues. While the majority of participants did not associate these feelings with specialist weight management services, a couple reported feeling nervous and disappointed with themselves before returning for their review appointment as they felt they had not reached agreed upon goals.

*“From the minute I left the appointment with my GP*, *I dreaded having to go back as he made me feel worthless*. *He said as I was going out the door*, *not having mentioned my weight for the whole time I was with him*, *we should have talked about your weight*, *it’s time for you to get moving and get that weight down or else you’ll be in trouble” (s14*, *PC)*

*“Since I have changed GP*, *I don’t mind going to the doctor*. *She is lovely and doesn’t judge me as soon as she sets eyes on me*. *Before this GP*, *I had a doctor that dismissed my weight concerns but at the same time attributed everything to my weight*. *He never took my seriously*. *So*, *no matter what was wrong*, *I avoided going back to him (s15*, *PC)*

*They made me feel so bad about my weight and you can imagine how emotional I already was*, *I never ever wanted to feel that way again*. *I never ever wanted to be pregnant again and never wanted to see those doctors again” (s10*, *SC (obstetrics))*

*Even in ___*, *I am sometimes nervous going back to my review appointment*, *especially if I haven’t kept to my eating plan or exercise plan and haven’t lost any weight*. *I think it’s more about me though*, *than the medical team*. *I don’t want to let them down as I feel they really care (s12*, *TC)*

*Escalation of Unhealthy Behaviours*. Escalation of unhealthy behaviours, associated with either personal ambivalence or shame, were often a response to being subjected to stigmatisation by a HCP. The most commonly reported harmful behaviour related to food where participants reported either ‘comfort’ or ‘binge’ eating. Some participants also reported that healthcare weight bias resulted in them taking a set backwards and reintroducing other harmful behaviours including smoking and physical inactivity.

*“I literally wouldn’t eat in front of anyone after being in hospital and being told how fat I was because I was so embarrassed*. *I was ashamed of putting anything into my mouth in public*. *But at the same time*, *behind closed doors*, *I was secretly eating more than ever before*. *I was comfort eating and putting on more and more weight” (s7*, *SC (orthopaedics))*

*“By the time I left the nurse in my GP’s surgery*, *I felt like a failure*. *She made me feel like it was all my fault that I was so fat*, *that I didn’t stick to the diet plan again and what’s the point*. *So off I go and I pulled into a petrol station and bought a family pack of chocolate and*, *two family bags of crisps and went home*, *sat on the couch and ate the whole lot*. *(s8*, *PC)*

*“The orthopaedic doctor told me I’d have to start walking an hour a day and loose 3 stone before my would contemplate surgery on my hip*, *I felt like screaming at him that my hip is way too painful to go out walking*. *Instead*, *to calm myself down*, *I lit a cigarette*, *the first cigarette in 6 months” (s5*, *SC (orthopaedics)*.

#### 3. Suggestions to address weight bias and stigma

Three sub-themes were identified as avenues to address bias and stigma and improve the healthcare experience of individuals living with obesity; empathy and equality, followed by healthcare professional education and finally, obesity clinical pathways.

*Empathy and Equality*. All participants expressed the feelings that their healthcare experiences, were, more often than not, lacking empathy, apart from consultations at the specialist weight management clinic. Participants stated it would really improve their clinical experience if HCPs would better try to understand how people living with obesity feel and use that understanding to guide their actions. In relation to equality, the majority of comments were around the feeling of unfairness and not being treated with the same level of professionalism or offered the same care as patients living without obesity.

*“I wish my doctor would see me as a person first*, *who has feelings*. *Not just as fat*. *If only he would ask me questions*, *try to understand how I got here*. *It’s not just black and white*, *it is way more complex than that” (s8*, *PC)*

*“My husband came away from his last doctor’s appointment delighted*. *Because the doctor explained to him all about his diabetes and suggested he sign up to a group run by the dietician and nurse in the surgery to help him manage it better*. *I diabetes and go to the same doctor and never has he suggested this group for me*. *I’m just told to lose weight (s13*, *PC)*

*“My advice to that surgeon is to drop the judgement as soon as you see me*, *leave it at the door and give me a chance*. *I’m here because I want to get rid of my knee pain and if you were a little bit more compassionate and positive*, *instead of talking at me*, *I might actually hear what you have to say (s7*, SC (orthopaedics))

*Healthcare Professional Education*. Participants’ main comments within this sub-theme related to their frustrations with HCPs in relation to what they termed as negative uninformed and unhelpful comments and information. Most of the interviewees felt that HCPs needed to be better educated about obesity and its complexity. Few clinicians provided anything more than a brief intervention around diet and physical activity and this was, more often than not, done, in passing, as they left the consultation. Many wondered if medical and nursing students learnt about obesity in college at all and its multifactorial / multicausal components. They also suggested getting patients living with obesity to talk to HCPs to explain their feelings of stigmatisation in many healthcare settings. The media, as a platform for education, was also mentioned as a source/cause of bias and stigma but also as a potential mediator of change.

*“All the nurses said was you need to cut down on high calorie food and do more exercise*. *She said this after taking my bloods when I was walking out the door*. *No plan*, *the same*, *same for the past 15 years*. *At this stage*, *I feel like saying to her*, *bigger picture …*. *It’s not that simple” (s2*, *PC)*

*“I think all doctors*, *nurses*, *physios*, *consultants*, *put obesity down to binge eating and not exercising*. *They think it is as simple as that (s3*, *non-specific)*

*“That’s another thing*, *when teaching students*, *maybe bring someone like me in*, *to give a talk about my experiences*, *how I feel and the negative effects that negativity has on patients (s10*, *non-specific)*

*“Maybe if all the images of obese people the media use on TV and in newspapers didn’t show fat people stuffing food into themselves*, *it might help to change peoples’ views*. *Even doctors must be influenced by those kind of images” (s14*, *non-specific)*

*Clinical Pathways*. The importance of a structured and timely clinical pathway for adults living with obesity with the opportunity to access specialist services, as required, was emphasised. The majority of participants felt they were in limbo for significant periods, sometimes years, in terms of their clinical weight management. All of those interviewed felt it was only when they become patients in the tertiary weight management clinic that they began to understand and gain some control over their obesity. Furthermore, all felt they should have been referred to this service at an earlier point on their journey. More than half of participants had to request that their GP refer them for specialist clinical input and this request had to be made more than once before it was processed.

*“There doesn’t seem to be a plan of action in place for people like me in the health service*. *I asked my GP about _____ more than five years before he actually referred me*. *It’s not OK to have to wait five years to see the experts*. *And even after I was referred*, *there was an 18-month waiting list*, *so it was nearly seven years after I first requested that I be referred to a specialist weight service*, *that I actually got an appointment” (s4*, *PC)*

*“If only I had been referred to the weight management clinic sooner*. *I’m not saying it would have made a huge difference to my weight loss*, *but it would definitely have helped me understand obesity and helped me accept myself for who I am much sooner*. *I’m a happier person for my experience at ____ hospital” (s11*, *TC)*.

## Discussion

This qualitative study, conducted in the Republic of Ireland, found that bias and stigmatisation is commonly experienced in the Irish healthcare system and is a significant threat to health and wellbeing issues for those living with obesity. Our results highlight the participants’ lived experiences in relation to weight bias and stigma, the consistent prejudice they have faced in both their primary and secondary healthcare encounters, how this has negatively impacted their overall healthcare and consequently, been detrimental to their physical and mental wellbeing. However, despite these overall negative healthcare experiences, participants were positive regarding the care received in weight management specific settings, with none having experienced weight bias and stigmatisation in this environment.

Experience of bias and stigma was characterised by poor quality communication, ambivalence towards medical concerns, refusal of care, and unsuitable physical environment in which healthcare was delivered. Consistent with previous findings, these issues resulted in participants describing overwhelming feelings of guilt, embarrassment, shame and worthlessness [13], known to result healthcare avoidance, appointment cancellation [31, 32] and refusal of care in the obstetric [33] and primary care [16] settings. Physical environment factors (e.g., furniture, medical equipment) were causes of discomfort and stress among our study participants, recognised intensifiers of this patient cohort’s discomfort and stress [34].

Our results reveal that people living with obesity are still being described using derogatory words such as ‘fat’ or ‘morbidly obese’, with this type of language known to be detrimental to their self-esteem, leading at times to patients seeking a new doctor [19, 35, 36]. Despite utilisation of people-first language when describing individuals with other medical conditions being a widely accepted norm, this does not seem to be the case for those afflicted by obesity. Expert stakeholders consider that changes to language used at the point of care could alleviate the stigma of obesity within the health-care system and support improved outcomes for both people living with obesity and for the health-care system [37]. A recent consensus statement [37] which addresses how people living with obesity wish to have their condition referred to, emphasised the negative connotations associated with the use of the word obese as an adjective and suggest that terms such as ‘carrying too much weight’ are broadly acceptable. Alternatively, the American Association of Clinical Endocrinology and American College of Endocrinology [38] have introduced the term ‘Adiposity-Based Chronic Disease (ABCD). This new diagnostic term for obesity explicitly identifies a chronic disease, alludes to a precise pathophysiologic basis, and avoids the stigmata and confusion related to the differential use and multiple meanings of the term "obesity."

As a result of the weight bias and stigma experienced, the study participants described how they avoided future healthcare consultations and more often than not, resorted to unhealthy behaviours, such as binge eating and increased sedentary time. These findings are substantiated in recent experimental and corelative research that found weight stigma, rather than obesity itself, can result in behaviours such as emotional overeating [39], and is strongly associated with exercise avoidance and lower levels of physical activity [40]. These behaviours inevitably result in weight gain over time and an increased feelings of failure and frustration. Furthermore, although research examining the physiological responses to weight stigma is in its infancy, preliminary results indicate that this stigma can trigger physiological stress responses that impair neuroendocrine control and contribute to increased adiposity, blood pressure and oxidative stress, higher levels of circulating C-reactive protein [41] and an elevated risk for cardiovascular and metabolic comorbidities [42, 43]. Paradoxically, experiencing weight bias and stigma from healthcare professionals whose role is to promote health, prevent disease and deliver patient centred healthcare [44], only serves to escalate the very psychological and physiological issues for which the patients have attended for help in managing to start with.

In terms of addressing weight bias and stigma in healthcare, participants had a number of ideas. Suggestions relating to communication and focus of care, were in accordance with positive experiences as patients’ attending the national weight management service, findings which are further endorsed by the available scientific evidence [34, 45, 46]. In the weight management service, healthcare providers expressed empathy, and care was delivered in an equitable and non-judgemental way. A collaborative rather than an educative communication approach was employed [34], where the participant felt they were being listened to [45, 46] and their opinion and needs was core to all medical decision-making [47].

Outside of the obesity specialist service, participants agreed that the majority of HCPs they encountered, particularly those working in primary care, obstetrics and orthopaedic clinics, irrespective of their profession, entirely attributed obesity to internal, controllable factors or personal choices. If and when HCPs discussed body weight with participants, they always focused on the oversimplified explanation of ‘calories in and calories out’ and physical inactivity, implying that body weight is completely controllable by voluntary decisions to eat less and exercise more, a conclusion that is entirely at odds with a definitive body of biological, clinical and social evidence developed over the past few decades [11]. At no stage, was there acknowledgment of the complexity of obesity and other influences on body weight such as genetics, epigenetics, the environment, societal factors and medications [48]. In light of these experiences, participants strongly suggested that healthcare practitioners and students be better educated and informed in terms of understanding that obesity is a complex, chronic condition with multiple aspects requiring a multi-faceted approach to its management.

While the most current research reveals obesity is not recognised as a priority in medicine [49], nursing [50] and physiotherapy education [51], inclusion of a formal obesity curriculum at entry and graduate level, that is co-designed with patients living with obesity, who have first-hand experience of healthcare weight bias and stigmatisation, should now be part of contemporary health and social care education. Moreover, in addition to training the next generation of HCPs, introduction of education resources for current HCPs, focusing on the complexity of obesity, the impact of weight stigma, environmental barriers and communication coaching, used alongside a ‘zero weight discrimination in healthcare’ policy, may be the first steps towards dispelling these negative attitudes [52].

Finally, participants highlighted the absence of a structured, timely clinical pathway for adults living with obesity. All participants felt they had no structured care plan, until they attended the weight management service. Most waited several years before being referred onwards by their general practitioner and, this only happened as a result of the patient themselves requesting such a referral. Furthermore, once they were referred, waiting time often exceeded two years.

Ireland, currently, only has two tertiary level weight management clinics and one centre for bariatric surgery, making access to such services geographically dependent and very limited in comparison to other European countries, most of which have a lower prevalence of obesity [53]. As of 2020, there are over 2000 adults on waiting lists for these weight management clinics and approximately 700 individuals waiting for bariatric surgery, some of whom have been waiting in excess of four years. Bariatric surgery rates annually equate to 18 per million of the population, compared to European averages ranging from 72 to 928 per million [54]. These statistics underline the necessity of an increase in obesity specific health services in a country where the prevalence of childhood and adult overweight and obesity is amongst the highest in Europe and internationally, with 8% of the adults aged > 50 years (92,573 people) meeting the criteria for bariatric surgery [54].

As important, however, as the tertiary weight management services is primary care. Primary care is the point of contact for most people seeking health services, and as gatekeepers of healthcare, primary care clinicians have enormous opportunities in the prevention of obesity and initiation of its management [55]. While acknowledging the change of mindset required, the educational resourcing and funding necessary to make obesity prevention and management a priority in this setting, recent European practical and patient centred guidelines for adult obesity management in primary care could potentially, provide a foundation on which to build a respectful and non-judgemental approach, free of bias and stigma [56].

As is customary with qualitative interview based research, this study has a number of limitations that should be considered when interpreting the results. We used a small, non-probability sample. This was decided in order to gather full and rich, personally ‘lived’ accounts of patients’ experiences of the Irish healthcare service in its entirety (primary, secondary and tertiary care). Since participation was voluntary, it is possible that this resulted in some bias of response. While we aimed to include an equal number of men and women in our study, the majority of volunteers were women, as is common in this area of research [15, 17–19, 33]. Future studies, should perhaps, focus on the male’s perspective. Notwithstanding these limitations, we believe that our data collection reached saturation point and our findings are context-specific, relevant and provide, for the first time, the perspective of patient and their experiences of obesity bias and stigma in the Irish healthcare setting.

While acknowledging the findings from these studies only provide the perspectives of a small number of patients, it is very clear that more needs to be done within Irish healthcare to dispel the negative attitudes towards patients living with obesity. A concerted effort by HCPs across clinical and educational levels is required to alleviate the harmful effects of obesity bias and stigma and facilitate a new narrative about obesity, that is coherent with modern scientific knowledge.

## Supporting information

S1 Fig Steps in the framework analysis method. (TIF)

S1 Table Standards for Reporting Qualitative Research (SRQR) 21 item checklist. (PDF)

S1 File Interview guide and questions. (PDF)
