# Supplementary figures and images for: A qualitative exploration of obesity bias and stigma in Irish healthcare; the patients’ voice
Source: PLoS One. 2021 Nov 29;16(11):e0260075. doi: 10.1371/journal.pone.0260075 (PMC8629268; doi:10.1371/journal.pone.0260075)

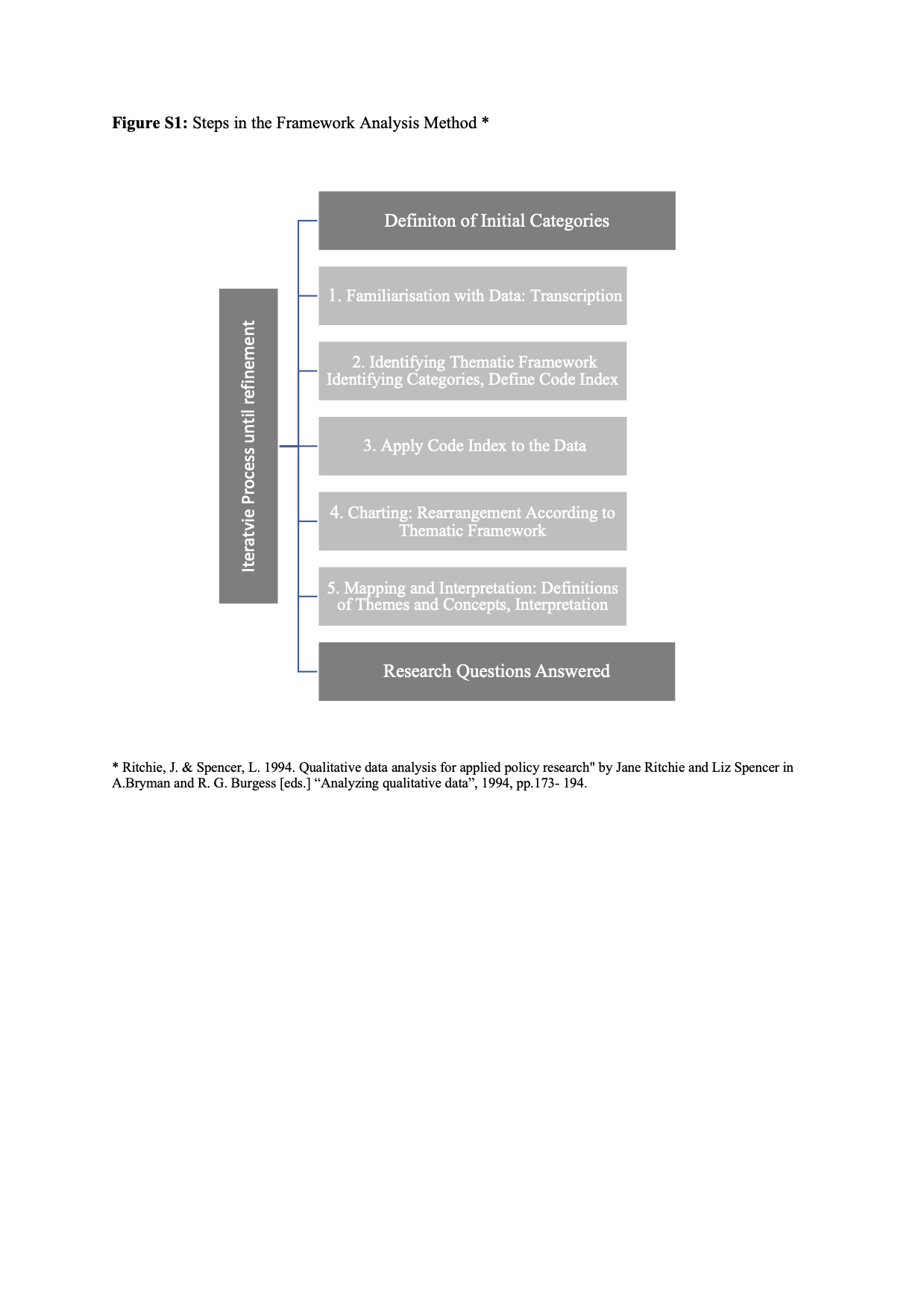

Supplement: S1 Fig — (TIF) [file pone.0260075.s001.tif]
