# Supplementary material for: A qualitative exploration of obesity bias and stigma in Irish healthcare; the patients’ voice
Source: PLoS One. 2021 Nov 29;16(11):e0260075. doi: 10.1371/journal.pone.0260075 (PMC8629268; doi:10.1371/journal.pone.0260075)
Supplement: S1 Table — (PDF) [file pone.0260075.s002.pdf]

Table S1: Standards for Reporting Qualitative Research (SRQR) 21 item checklist *

Number	Topic	Item	Included	Page
	**Title and Abstract**			
S1	Title	Concise description of the nature and topic of the study, identifying the study as qualitative or indicating the approach (e.g., ethnography, grounded theory) or data collection methods (e.g., interview, focus group) is recommended	✓	1
S2	Abstract	Summary of key elements of the study using the abstract format of the intended publication; typically included background, purpose, methods, results and conclusions	✓	2
	**Introduction**			
S3	Problem formulation	Description and significance of the problem/phenomenon studied; review of relevant theory and empirical work; problem statement	✓	3
S4	Purpose or research question	Purpose of the study and specific objectives or questions	✓	4
	**Methods**			
S5	Qualitative approach and research paradigm	Qualitative approach (e.g., ethnography, grounded theory, case study, phenomenology, narrative research) and guiding theory if appropriate ; identifying the research paradigm (e.g., postpositivist, constructivist/interpretivist) is also recommended; rationale	✓	4
S6	Researcher characteristics and reflexivity	Researchers' characteristics that may influence the research, including personal attributes, qualifications/experience, relationship with participants, assumptions and/or presuppositions; potential or actual interaction between researchers' characteristics and the research question, approach, methods, results and/or transferability	✓	4
S7	Context	Setting/site and salient contextual factors; rationale	✓	5
S8	Sampling strategy	How and why research participants, documents, or events were selected; criteria for deciding when no further sampling was necessary (e.g., sampling saturation); rationale	✓	5
S9	Ethical issues pertaining to human subjects	Documentation of approval by an appropriate ethics review board and participant consent, or explanation for lack of thereof; other confidentiality and data security issues	✓	6
S10	Data collection methods	Types of data collected; details of data collection procedures including (as appropriate) start and stop dates of data collection and analysis, iterative process, triangulation of sources/methods, and modification of procedures in response to evolving study findings; rationale	✓	5&6

S11	Data collection instructions and technologies	Description of instruments (e.g., interview guides, questionnaires) and devices (e.g., audio recorders) used for data collection: if/how the instrument(s) changed over the course of the study	✓	6
S12	Units of study	Number and relevant characteristics of participants, documents or events included in the study; level of participation (could be reported in results)	✓	5
S13	Data processing	Methods for processing data prior to and during analysis, including transcription, data entry, data management and security, verification of data integrity, data coding and anonymisation/deidentification of excerpts	✓	6 & Figure S1
S14	Data analysis	Process by which inference, themes, etc were identified and developed, including the researchers involved in data analysis; usually references a specific paradigm or approach; rationale	✓	6 & Figure S1
S15	Techniques to enhance trustworthiness	Techniques to enhance trustworthiness and credibility of data analysis (e.g., member checking, audit trail, triangulation); rationale	✓	6
	**Results/findings**			
S16	Synthesis and interpretation	Main findings (e.g., interpretations, inferences, and themes); might include development of a theory or model, or integration with prior research or theory	✓	6-13
S17	Links to empirical data	Evidence (e.g., quotes, field notes, text excerpts, photographs) to substantiate analytic findings	✓	6-13
	**Discussion**			
S18	Integration with prior work, implications, transferability and contribution to the field	Short summary of main findings; explanation of how findings and conclusions connect to, support, elaborate on, or challenge conclusions of earlier scholarship; discussion of scope of application/generalisability, identification of unique contribution(s) to scholarship in a discipline or field	✓	14-17
S19	Limitations	Trustworthiness and limitations of findings	✓	17-18
	**Other**			
S20	Conflicts of Interest	Potential sources of influence or perceived influence on study cohort and conclusions; how these were managed	✓	^^
S21	Funding	Sources of funding and other support; role of funders in data collection interpretation and reporting	✓	^^

* O'Brien BC, Harris IB, Beckman TJ, Reed DA, Cook DA. Standards for reporting qualitative research: a synthesis of recommendations. Academic Medicine 2014; 89(9): pp. 1245-1251
^^ Included at submission (Financial Disclosure and Competing Interests sections)
