# Supplementary material for: A qualitative exploration of obesity bias and stigma in Irish healthcare; the patients’ voice
Source: PLoS One. 2021 Nov 29;16(11):e0260075. doi: 10.1371/journal.pone.0260075 (PMC8629268; doi:10.1371/journal.pone.0260075)
Supplement: S1 File — (PDF) [file pone.0260075.s003.pdf]

SEMI-STRUCTURED INTERVIEW GUIDE

A qualitative exploration of obesity bias and stigma in Irish healthcare: the patient's voice

Welcome
Thank you for taking part in this study and for agreeing to be interviewed

Introductions
I know we have already met briefly but by way of introduction;
My name is ….., I am a ….., My background is in …..
My colleagues and I are very interested in the subject of weight bias and stigma in healthcare, the topic of today's interview and really interested in hearing your thoughts on the subject. If it is OK with you, I will be recording the interview as I won't remember everything we discuss and we want to capture as much of what you say as possible.

Structure
The interview will take a maximum of 60 minutes and will be structured around a number of questions. The questions I will guided by today were composed using existing research on obesity bias and stigma. Please just answer as honestly and openly as you can. More often than not, a discussion will ensue from a question – that's fine and exactly what we are aiming for

Ground Rules

- There are no right or wrong answers. Each individual person's experiences and opinions are really important. Speak up whether you agree or disagree. We want to hear your personal perspective.
- We want you to feel comfortable sharing positives and negatives. The more open and honest you can be, the better for the research study.

QUESTION GUIDE

- Can you tell me about some of interactions you have had with a doctor or nurse or physiotherapist (expand healthcare professional (HCP) list as required) that involved anything to do with your body size?
- Can you describe how you felt after your last appointment with a HCP ? (Is this the norm ….)
- Did you have any sense of how the HCP found the interaction (satisfying or unsatisfying)?
- Do you have any suggestions about how the experiences you described could have been made more positive for you as a patient?
- In your opinion, are there any really obvious ways of eliminating weight bias and stigma in healthcare?

Possible Prompts
- Do you remember any words or phrases or how things were 'put'?
- Did you feel that the reason you went to see the HCP in the first place was addressed?
- Was there anything about the physical environment (e.g., equipment/furniture) in this clinic that you noticed your body size? For example, did it make you feel comfortable or uncomfortable?
- Can you expand on that please

I think that's basically everything I wanted to ask. Do you have anything else you would like to raise or final thoughts you have had? Anything you think that might be relevant that I haven't asked? Or anything you would like to add.…

Conclusion
I'll finish by saying thank you very much for sharing your thoughts and expertise with me.
It is much appreciated …
